# Mutation analysis of the ferritin L-chain gene in age-related cataract

**Published:** 2010-11-24

**Authors:** Nurit Assia, Nitza Goldenberg-Cohen, Gideon Rechavi, Ninette Amariglio, Yoram Cohen

**Affiliations:** 1 Sackler School of Medicine, Tel Aviv University, Tel Aviv, Israel; 2 The Pediatric Eye Research Laboratory, Felsenstein Medical Research Center, Tel Aviv University Petach Tikva, Israel; 3 Ophthalmology Department, Pediatric Division, Schneider Children’s Medical Center of Israel, Petach Tikva, Israel; 4 Cancer Research Center, Sheba Medical Center, Tel-Hashomer, Sackler Faculty of Medicine, Tel Aviv University, Tel Aviv, Israel; 5 Department of Obstetric and Gynecology, Sheba Medical Center, Tel Hashomer, Israel

## Abstract

**Purpose:**

To investigate whether acquired somatic mutations in the iron response element of the ferritin L-chain gene account for the age-related cataract.

**Methods:**

The 15 most prevalent point mutations causing hereditary hyperferritinemia cataract syndrome (HHCS) were screened in patients with age-related cataract using MALDI-TOF Mass Spectrometry. DNA samples were obtained from the lens capsules of patients following cataract surgery, and subjected to PCR amplification. Products were analyzed by a Sequenom® mass spectrometer, and classified as a mutation or wild type according to molecular weight. For a positive control, L-ferritin G32T mutation detected by direct sequencing in 3 members of an Israeli family known to be affected by HHCS was used.

**Results:**

DNA samples were isolated from the lens capsules of 90 patients, mean age 73.86, and screened for L-ferritin mutations. While the G32T mutation was detected in all 3 positive control cases, all other patients were negative for the 15 mutations.

**Conclusions:**

Somatic mutations in the iron response elements (IRE) of the L-ferritin gene are infrequent in the age-related cataract. The role of L-ferritin genetic variations in the pathogenesis of age-related cataract is yet to be explored.

## Introduction

Cataract, or opacity of the intraocular lens, is the most common cause of curable blindness worldwide. It is treated by surgical removal of the native lens, and its replacement by an artificial one. Cataracts may be classified by etiology into the age-related cataract, the congenital and juvenile cataract, or the cataract secondary to trauma, intraocular diseases, systemic diseases, or exposure to noxious agents such as radiation or certain drugs. The age-related cataract is by far the most prevalent type of cataract, and is attributed to changes in the lens structure and composition due to oxidative damage which occurs with aging [1,2]. The congenital cataract is rare, and reflects mainly genetically-caused developmental alterations in the lens and surrounding ocular tissues [3]. The discovery of a broad variety of genes important for eye and lens development has progressed greatly in the recent years [3]. Nevertheless, there still remains a long list of mutations to be characterized, indicating a broad genetic heterogeneity which clinicians simply refer to as a “cataract” [3].

Serum ferritin is normally a marker of iron overload. Ferritin genes are sited at chromosomes 19 and 11. Regulation of ferritin synthesis involves the interaction between an iron regulatory protein (IRP) and part of the ferritin mRNA designated the iron regulatory elements (IREs). A disorder of ferritin synthesis resulting in hyperferritinemia in the absence of iron overload has been described [4]. Hereditary hyperferritinemia cataract syndrome (HHCS), a syndrome of early-onset cataract and hyperferritinemia, is caused by germline mutations in the iron response element (IRE) of the ferritin L-chain (L-ferritin) gene suggesting that the ferritin metabolism plays a crucial role in the pathogenesis of cataract [5-8]. HHCS is an autosomal dominant disease caused by mutations in the IRE of the L-ferritin gene [9]. It is not unusual to observe increased levels of ferritin in apparently healthy patients [10]. HHCS is a genetic condition characterized by a bilateral nuclear cataract of early onset. This overexpression of the L-chain in the lens causes bilateral nuclear cataracts, which are the specific sign of this syndrome [10]. Cataract morphology in HHCS is highly distinctive. Ophthalmological assessment of patients with hyperferritinemia, even when asymptomatic, may lead to a correct diagnosis of HHCS [11].

Ferritin is an iron storage protein which binds excess iron in cells. It consists of 24 heavy (H-chain, 21 kDa) or light (L-chain, 19 kDa) subunits, in ratios varying in the different tissues [12,13]. Ferritin synthesis is tightly regulated by plasma iron levels [14]. This regulation is achieved by the high-affinity interaction between non-coding mRNA stem-loop structures known as IREs and cytoplasmic mRNA-binding proteins known as IRPs [14]. The binding of IRP to IRE in the 5′ untranslated regions (UTR) of ferritin blocks translation and thus inhibits ferritin synthesis [12]. It has been demonstrated that single or double point mutations or deletions in the stem-loop structure of the IREs of the ferritin L-subunit gene (chromosome 19q13.1) are responsible for the upregulation of ferritin [10]. When intracellular iron levels are high, a cluster of [4Fe-4S] binds to IRPs, preventing their binding to the IRE. This enables the translation of ferritin which acts to sequester and store free excess iron. In iron repletion states there is little interference in IRP and IRE interaction; ferritin translation is blocked and free iron levels rise [9].

HHCS arises from various point mutations or deletions within the IRE of the L-ferritin gene, leading to the overexpression of the L-chains in ferritin [9,13]. Ever since families with HHCS were first described in 1995, almost 30 germline mutations in the L-ferritin gene have been reported [15]. However, to this day, the mechanism by which ferritin mutations lead to cataract remains unclear.

Gorlaska et al. [13] showed the accumulation of L-chain-enriched ferritin associated with cytoplasmic inclusion bodies. The formation of inclusion bodies in older lens epithelial cells, indicates the possible involvement of cytoplasmic L-chain-enriched ferritin aggregates in the formation of age-related cataract [13]. Several studies indicate that ferritin and more specifically ferritin H-chains, offers protection against UV radiation. The same group [16] described the effects of UV radiation on canine lens epithelial cells following transfection with an expression vector containing the coding sequence of either L- or H-chain cDNA. UV radiation reduced the cell numbers of L-chain transfectants by half whereas H-chain transfectants were protected [16]. As UV radiation is a known risk factor for the development of cataract, overexpression of L-chains as a result of mutations may be responsible for the increased susceptibility for the formation of a cataract.

No relevant symptoms other than visual impairment were found to be associated with the HHCS syndrome [9]. A marked phenotypic variability was observed, and similarly, serum ferritin levels varied substantially within subjects sharing the same mutation [9]. Well defined lens opacities were not detectable either at birth or at 1 year of HHCS newborn [9]. In a previous study, the lens ferritin content was analyzed in two subjects who underwent cataract surgery at different ages, with different cataract morphology; values were similar and about 1,500 fold higher than in controls [9]. These observations suggested that in HHCS the cataract is not necessarily congenital and that in addition to the IRE genotype, other genetic or environmental factors may modulate the phenotype, especially the severity of the cataract.

Based on the hypothesis that the ferritin metabolism plays a crucial role in the pathogenesis of cataract, we theorized that acquired somatic mutation in the lens capsular DNA may account for age-related cataract. Such mutations could possibly follow exposure to mutagenic insults/oxidative stress such as UV radiation. Our assumption was that alterations of the L-ferritin gene in lens capsular cells may lead to local changes in the ferritin metabolism, which could cause instability of lens structures and ultimately cataract.

In this study we investigate the prevalence of 15 point mutations within the IRE of the L-ferritin gene previously shown to cause HHCS, in DNA obtained from lens capsules of senile cataract extracted in cataract surgery.

## Methods

### Patients

Ninety surgically excised anterior lens capsules and lens material were collected with informed consent during routine cataract surgery in Rabin Medical Center (Petach Tikva, Israel) between 2006 and 2007 with Institutional and National Genetic Review Board approval and were used for analysis. Patients’ records were reviewed for age, sex and cataract type.

### DNA isolation

The capsules and lens material containing epithelial cells were suspended in 5 ml conservation medium (PBS) and kept in 4 °C until isolation of genomic DNA. DNA was extracted using standard SDS/proteinase K digestion followed by phenol-chloroform extraction and ethanol precipitation. DNA concentrations were estimated by spectrophotometric (OD) quantification and diluted to 2.5–25 ng/μl.

### Mutation detection

Scanning of mutations in the L-ferritin 5′ UTR regulatory sequence was performed using the MALDI-TOF Sequenom (Sequenom, San Diego, CA) platform. We constructed and validated a molecular assay that detects the 15 most common point mutations that have been reported in the literature (Table 1).

**Table 1:** Point mutation in the L-ferritin 5’UTR.

**Mutation**	**Described by**
T22G	Cazzola et al. [20]
G41C	Cremonesi et al. [21]
C18T	Cazzola et al. [20]
C10T	Cremonesi et al. [15]
C14G	Cremonesi et al. [21]
C16T	Cremonesi et al. [15]
C90T	Cremonesi et al. [15]
G51C	Camaschella et al. [22]
G32T/C/A	Martin et al. [23], Kato & Casella [24], Cazzola et al. [20], Cicilliano et al. [25]
C33T	Balas et al. [26]
C36G	Cremonesi et al. [15]
C36A	Mumford et al. [27]
A37G	Cremonesi et al. [15]
C39T	Balas et al. [26]
A40G/C	Beaumont et al. [5]

### Assay design

Simplex SNP assays were designed using Sequenom‘s MassARRAY Assay Design V3.1 software (Sequenom). The assay design and the list of amplification and extension primers are provided in Table 2 and Table 3.

**Table 2:** Assay design.

**Mutation ID**	**Amplicon length**	**Ext. primer direction**	**Ext. primer mass**	**First allele call**	**First allele mass**	**Second allele call**	**Second allele mass**	**Third allele call**	**Third allele mass**	**Fourth allele call**	**Fourth allele mass**
T22G	111	R	5596.7	G	5869.9	T	6183.1				
G41C	120	F	5111.3	C	5384.5	G	5728.7				
C18T	111	R	5206.4	T	5503.6	C	5808.8				
C10T	111	F	5138.3	C	5411.5	T	5755.7				
C14G	111	F	5178.4	C	5451.5	G	5780.8				
C16T	111	F	5178.4	C	5451.5	T	5795.8				
C90T	109	F	5052.3	C	5325.5	T	5933.9				
G51C	114	F	5489.6	C	5762.8	G	6445.2				
G32T/C	111	R	5507.6	G	5780.8	A	5795.8	T	5804.8	C	6134
C33T	111	R	5467.6	T	5764.8	C	6070				
C36G	113	F	5174.4	C	5447.5	G	5800.8				
C36A	113	F	5174.4	C	5447.5	A	6387.2				
A37G	114	F	5134.3	A	5431.5	G	5760.7				
C39T	120	R	5105.3	T	5402.5	C	5722.7				
A40G/C	120	F	5391.5	C	5664.7	A	5688.7	G	6338.1		

**Table 3:** Primer list.

**Mutation ID**	**1st-PCR primer**	**2nd-PCR primer**	**Extension primer**
L-ferritin-T22G	ACGTTGGATGGATCTGTTCCGTCCAAACAC	ACGTTGGATGATAAAAGAAGCCGCCCTAGC	TGTTGAAGCAAGAGACAG
L-ferritin-G41C	ACGTTGGATGTAAAAGAAGCCGCCCTAGCC	ACGTTGGATGAGAGTCCCCGGATCTGTTC	TGTCTCTTGCTTCAACA
L-ferritin-C18T	ACGTTGGATGGATCTGTTCCGTCCAAACAC	ACGTTGGATGATAAAAGAAGCCGCCCTAGC	AAGCAAGAGACAGACCC
L-ferritin-C10T	ACGTTGGATGATAAAAGAAGCCGCCCTAGC	ACGTTGGATGGATCTGTTCCGTCCAAACAC	GTCCCCTCGCAGTTCGG
L-ferritin-C14G	ACGTTGGATGATAAAAGAAGCCGCCCTAGC	ACGTTGGATGGATCTGTTCCGTCCAAACAC	CCTCGCAGTTCGGCGGT
L-ferritin-C16T	ACGTTGGATGATAAAAGAAGCCGCCCTAGC	ACGTTGGATGGATCTGTTCCGTCCAAACAC	TCGCAGTTCGGCGGTCC
L-ferritin-C90T	ACGTTGGATGACAGTGTTTGGACGGAACAG	ACGTTGGATGATGGTCCCGGAGGTTGCAAG	TCCAGCCTCCGACCGCC
L-ferritin-G51C	ACGTTGGATGGGTCTGTCTCTTGCTTCAAC	ACGTTGGATGTTGCAAGCGGAGAGGAAATC	CTTCAACAGTGTTTGGAC
L-ferritin-G32T/C	ACGTTGGATGGATCTGTTCCGTCCAAACAC	ACGTTGGATGATAAAAGAAGCCGCCCTAGC	GTCCAAACACTGTTGAAG
L-ferritin-C33T	ACGTTGGATGGATCTGTTCCGTCCAAACAC	ACGTTGGATGATAAAAGAAGCCGCCCTAGC	CGTCCAAACACTGTTGAA
L-ferritin-C36G	ACGTTGGATGATAAAAGAAGCCGCCCTAGC	ACGTTGGATGCGGATCTGTTCCGTCCAAA	GGGTCTGTCTCTTGCTT
L-ferritin-C36A	ACGTTGGATGATAAAAGAAGCCGCCCTAGC	ACGTTGGATGCGGATCTGTTCCGTCCAAA	GGGTCTGTCTCTTGCTT
L-ferritin-A37G	ACGTTGGATGATAAAAGAAGCCGCCCTAGC	ACGTTGGATGCCGGATCTGTTCCGTCCAAA	GGTCTGTCTCTTGCTTC
L-ferritin-C39T	ACGTTGGATGAGAGTCCCCGGATCTGTTC	ACGTTGGATGTAAAAGAAGCCGCCCTAGCC	TGTTCCGTCCAAACACT
L-ferritin-A40G/C	ACGTTGGATGTAAAAGAAGCCGCCCTAGCC	ACGTTGGATGAGAGTCCCCGGATCTGTTC	TCTGTCTCTTGCTTCAAC

### Assay outline

384-well plates containing 2.5–25 ng of DNA in each well were subjected to PCR amplification reactions following the specifications of Sequenom (See below, PCR conditions section). After PCR, shrimp alkaline phosphatase (Sequenom) was added to the samples to dephosphorylate excess dNTPs to prevent their future incorporation and interference with the primer extension assay. Allele discrimination reactions were conducted by adding the extension primer assay, DNA polymerase, and a mixture of dNTPs and ddNTPS to each well. MassExtend clean resin (Sequenom) was added to the mixtures to remove extraneous salts that could interfere with MALDI-TOF analysis.

Genotyping was determined by spotting an aliquot of each sample onto a 384 SpectroChip (Sequenom), which was subsequently analyzed by the chip-based matrix-assisted laser desorption time-of-flight (MALDI-TOF) mass spectrometer (Sequenom). With this technique, each spotted sample is analyzed using laser-mediated desorption and ionization of the extended oligonucleotide product. This results in the acceleration of the oligonucleotide toward a detector. The velocity of the sample is proportional to oligonucleotide length. As a result, time from laser-mediated desorption and ionization to detector signaling (time of flight – TOF) is directly correlated with oligonucleotide mass.

The resulting spectra were converted to meaningful genotype data using SpectroTYPER-RT software (Sequenom), which interprets the spectral output based on information for expected allele-specific oligonucleotide lengths generated during the assay design phase(see above). Since the sensitivity of the system was previously determined to be limited to detecting only mutations composing over 1%–3% of the analyzed DNA [17], results were reviewed thoroughly.

### PCR conditions

PCR amplifications were performed in standard 384-well plates, a 5 µl final volume containing 10 ng of template DNA, 0.1 U of Taq polymerase (HotStarTaq, Qiagen, Valencia, CA), 0.2 mM of each dNTP, 200 nmol of the appropriate primer (Forward and Reverse), 1 mM MgCl_2_ and 1× HotStar buffer.

PCR thermal cycling was performed in an ABI-9700 instrument for 15 min at 95 °C, followed by 4 cycles of 20 s at 95 °C, 30 s at 65 °C and 60 s at 72 °C, 4 cycles of 20 s at 95 °C, 30 s at 58 °C, and 60 s at 72 °C and 38 cycles of 20 s at 95 °C, 30 s at 53 °C and 60 s at 72 °C. This was followed by incubation with 0.3 U Shrimp alkaline phosphates in total volume of 7 µl for 20 min at 37 °C and 5 min at 85 °C.

The MassEXTEND® (Sequenom) assay was conducted in 9 µl final volume containing 1 µM extension primer, 0.2 µl of termination mix (50 nM each of ddA, dG, ddT and ddC) and 1.25 U ThermoSequenase (SequenomA) in 0.22× PCR buffer. The cycling conditions were: incubation for 2 min at 94 °C followed by 99 cycles of 5 s at 94 °C, 5 s at 52 °C and 5 s at 72 °C. Following this step, 3 µg MassEXTEND® cleanup resin (Sequenom) and 16 µl DDW were added to remove extraneous salts.

Samsung nanodispenser was used to apply 15 nl of extension products from each well of the sample plate onto the SpectroChip (Sequenom). Mass spectra were recorded on a Bruker Biflex MALDI-TOF mass spectrometer operated in the linear mode, and were analyzed by MassARRAY Typer software (Sequenom).

### Result validation (positive control)

Results were validated by performing MALDI-TOF genotyping of 3 family members positive for the G32T mutation previously detected by direct DNA sequencing.

## Results

Ninety patients (40 men and 50 women) participated in the study. The patients had undergone cataract surgery in Rabin Medical Center (Petah Tikva, Israel) between the years 2006 and 2007. Patients were 54–91 years old at the time of surgery (mean age 73.86).

**Figure 1:**
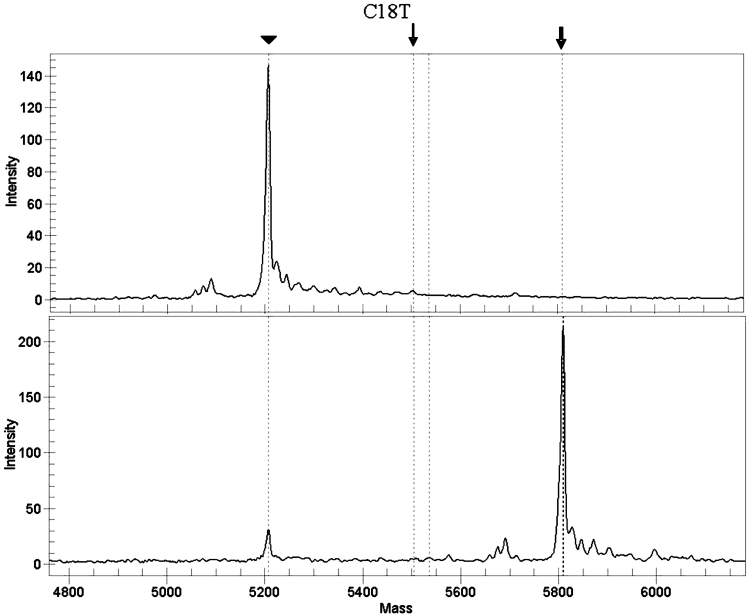
Representative spectra of the C18T assay. The arrowhead indicates the unextended primer. The bold arrow indicates the wild type allele (C18). The thin arrow indicates the potential location of the mutated allele (C18T). Top panel: A template-free control sample. Bottom panel: Wild type allele.

The DNA samples were screened for 15 point mutations in the IRE of the L-ferritin gene. One assay (testing for the G51C mutation) failed constantly and was excluded from the study. Figure 1 shows a representative spectra of the C18T assay.

**Figure 2:**
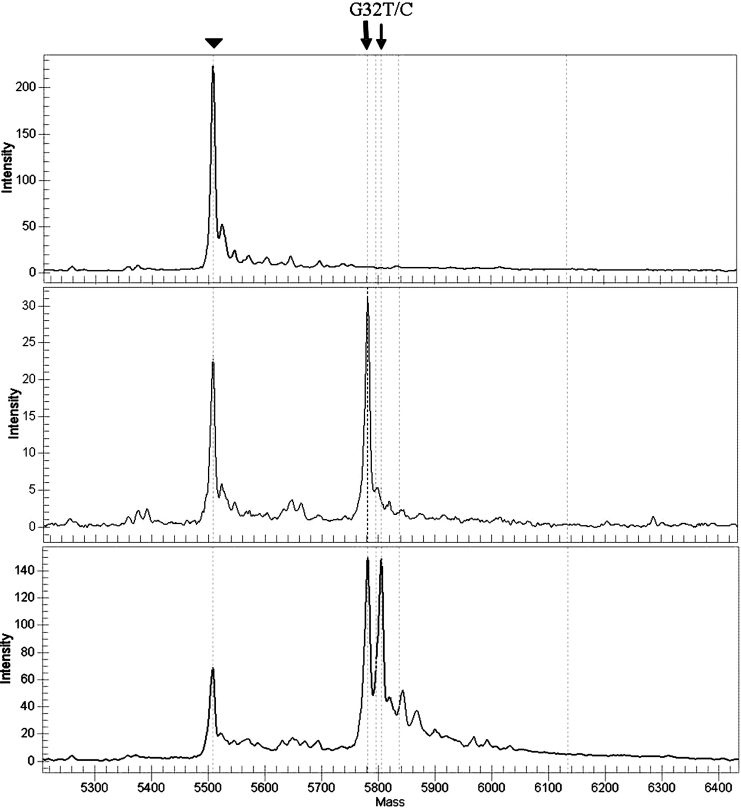
Result validation. The G32T mutation was detected by direct sequencing in three Israeli family members with HHCS. DNA samples from all three were also analyzed using the MALDI-TOF technique and were used as positive controls for the G32T mutation. The arrowhead indicates the unextended primer. The bold arrow indicates the wild type allele. The thin arrow indicates the mutated allele. Top panel: A spectrum from a template-free sample (negative control). Middle panel: A spectrum from a patient negative for the mutation. Bottom panel: A spectrum from a subject with HHCS (positive control).

**Figure 3:**
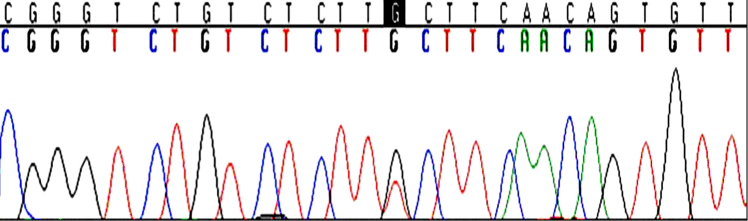
The chromatogram of a patient harboring the G32T mutation. The black square marks the position of the mutation.

DNA samples from 3 HHCS patients positive for the G32T germline mutation, as determined by direct sequencing, were used as positive controls for the G32T/C assay. These samples were analyzed using the MALDI-TOF technique and the G32T mutation was detected in all 3 cases (Figure 2). The corresponding chromatogram from one of the patients is shown in Figure 3.

The 15 most common HHCS-related somatic mutations were not detected in our study group.

## Discussion

Discovering an association between acquired somatic mutations and the development of age-related cataract may have meaningful clinical implications. Studying such mutations could lead to a better understanding of the underlying mechanisms of cataract, and perhaps aid in preventing rather then treating one of the world’s most common disorders in adults.

Based on the hypothesis that a disturbance in normal ferritin metabolism causes lens opacification in patients with HHCS, we screened adult age-related cataract tissue for the 15 most prevalent HHCS point mutations described in the literature.

In this study, no mutations were detected in any of the 15 sites screened. However, these mutations represent only one genetic syndrome of the different congenital disorders which present with bilateral cataract. Although this study has not shown a connection between acquired mutations in the L-ferritin gene and age related cataract, such connections may still exist with other genes already known to be involved in congenital cataract.

It has been suggested in many studies that both genetics and environment play a role in the pathogenesis of cataract. Hammond et al. [18] reported in 2001 the relative contribution of genes, environment and age in the evolution of cataract in a study of pairs of monozygotic and dizygotic twins. The main contribution of the genetic factor was for cortical cataract. Francis et al. [19] hypothesized in 1999 that age-related cataract involves “mild” mutations in the same genes involved in congenital cataract, except that in the congenital form the same genes are more severely affected.

This study explores a different model to explain cataract formation. As opposed to other studies which focused on the late expression of congenital genetic factors, the hypothesis of this study was that the genetic changes may have been acquired over the years.

For genetic variation screening we chose to use the Sequenom® technology which allows for large-scale screening of mutations. Applying MALDI-TOF mass spectrometry enabled a relatively simple genetic analysis of capsular DNA. The analysis was performed by the system, which interpreted the particles’ time of flight and determined whether alleles are wild type or mutant according to data previously generated in the Assay Design phase. System error rates were minimal, and most were false positive rather than preventing recognition of a true mutation.

In conclusion, no mutations were detected in the study in the IRE region of the L-ferritin gene. However, this does not exclude the possibility of mutations in other regions in the L-ferritin gene, H-ferritin gene or other genes that may contribute to cataract formation. If such mutations will be discovered, it may be possible to offer patients intervention at earlier stages of the disease, and offer treatment targeting the affected genes, that may delay or prevent the need for surgery.
